# PP2C-like Promoter and Its Deletion Variants Are Induced by ABA but Not by MeJA and SA in *Arabidopsis thaliana*

**DOI:** 10.3389/fpls.2016.00547

**Published:** 2016-05-03

**Authors:** Purva Bhalothia, Chetna Sangwan, Anshu Alok, Sandhya Mehrotra, Rajesh Mehrotra

**Affiliations:** ^1^Department of Biological Sciences, Birla Institute of Technology and SciencesPilani, India; ^2^Department of Biotechnology, National Agri-Food Biotechnology InstitutePunjab, India

**Keywords:** abscisic acid, ACGT *cis* element, abiotic stress, *cis* regulatory elements, gene regulation

## Abstract

Gene expression is mediated through interaction between *cis* regulatory elements and its cognate transcription factors. *Cis* regulatory elements are defined as non-coding DNA sequences that provide the binding sites for transcription factors and are clustered in the upstream region of genes. ACGT *cis* regulatory element is one of the important *cis* regulatory elements found to be involved in diverse biological processes like auxin response, salicylic acid (SA) response, UV light response, ABA response and jasmonic acid (JA) response. We identified through *in silico* analysis that the upstream region of protein phosphatase 2C (*PP2C*) gene has a distinct genetic architecture of ACGT elements. In the present study, the activation of the full length promoter and its deletion constructs like 900 base pair, 500 base pair, 400 base pair and NRM (Nathji Rajesh Mehrotra) were examined by stable transformation in *Arabidopsis thaliana* using β-*glucuronidase* as the reporter gene. Evaluation of deletion constructs of *PP2C*-like promoter was carried out in the presence of phytohormones like abscisic acid (ABA), SA and JA. Our result indicated that the full length and 900 base pair promoter-reporter constructs of PP2C-like promoter was induced in response to ABA but not to methyl jasmonate and SA.

## Introduction

Abiotic and biotic stresses drastically affect the plant growth and productivity. In response to these challenges plants adapt themselves through various mechanisms which are controlled by the molecular, physiological and cellular processes (Mehrotra et al., 2014). Phyto hormones such as ABA, SA, JA, and ethylene (ET) tend to play a pivotal role in helping plants to adapt themselves to abiotic and biotic adversities (Fujita et al., 2006). Various molecular approaches can be used to improve the plant growth and productivity, plant genetic engineering being one of the most important strategy. The CaMV-35S, a constitutive promoter is extensively used in plant genetic engineering. Over expression of stress responsive genes under the control of such constitutive promoters is of great importance. However, the potential advantage is compensated by stunted growth, delayed germination, seed dormancy, and photobleaching. It has been shown that the constitutive expression of stress inducible gene, OsbZIP71 by CaMV35S promoter resulted in delayed flowering time (Liu et al., 2014). Also DREB/CBF genes under the regulation of CaMV35S promoter have shown improved tolerance to salt, drought and cold stress but along with various (growth and developmental) abnormalities (Jaglo-Ottosen et al., 1998). Usage of inducible promoters is a plausible solution to such problems. To regulate OsbZIP71, Liu et al. (2014) used *rd*29A gene promoter, i.e., an inducible promoter from *Arabidopsis* instead of a constitutive promoter. Similarly, drought resistance was enhanced with the expression of LeNCED-1 gene in petunia under the control of an inducible promoter *rd*29A. Use of such promoters is an effective genetic engineering strategy (Mehrotra et al., 2011) with minimal side effects (Estrada-Melo et al., 2015).

Promoter of the ABA dependent stress responsive genes have ABA responsive element (ABRE) with ACGT as a core sequence (Foster et al., 1994). It has been reported that the expression of ABA-responsive genes require more than one ABRE or a combination of an ABRE and a coupling element (CE) for an optimal gene expression (Marcotte et al., 1989; Hobo et al., 1999). Mehrotra and Mehrotra (2010) showed that two ACGT elements with a spacer of 25 nucleotides are induced in response to ABA. However, when separated by five nucleotides, they are induced by SA in transgenic tobacco plants. The genome wide analysis of *A. thaliana* revealed the frequency of occurrence of two ACGT motifs separated by a spacer of 25 bp to be 62 while for 5 bp, the occurrence was 72 (Mehrotra et al., 2011).

One such promoter (accession number AT5G59220) is PP2C-like promoter located at chromosome number 5 in *A. thaliana*. Genetic architecture of PP2C-like promoter includes three ACGT elements in close vicinity. Two adjacent ACGT elements are separated by 30 nucleotides, followed by another set of ACGT motifs separated by five nucleotides. PLACE (Plant *cis*-acting regulatory DNA elements) and Plant Care database were used to analyze an array of hormone inducible *cis* regulatory elements in PP2C-like promoter. In the present study, PP2C-like promoter was cloned and deletion constructs were prepared and transformed in *A. thaliana* plants through floral dip method (Clough and Bent, 1998) to check their effect on the expression of β-*glucuronidase* reporter gene cloned downstream of it.

PP2C-like promoter comprise both biotic stress and abiotic stress response elements in close proximity which provides an advantage over the constitutive promoters and it could be used as an inducible promoter.

We cloned PP2C promoter from *A. thaliana* and fused it with β*-glucuronidase* (*UidA*) gene in a binary vector pBI101. Five promoter – reporter constructs were prepared using PP2C-like promoter which were then transformed into *Agrobacterium*. Finally *Arabidopsis* plants were transformed via floral dip method. These transgenic plants were then screened by kanamycin selection and PCR. The transgenic plants were treated with ABA, MeJA, and SA hormones at different time intervals. In this study the expression of full length and 900 bp deletion constructs have shown maximum expression in the presence of ABA.

## Materials and Methods

**FIGURE 1:**
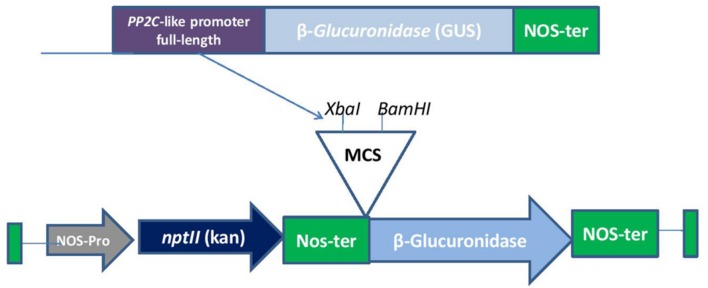
**A diagrammatic representation of full-length construct in pBI101**.

The sequence of PP2C-like promoter was obtained from TAIR database and *cis* regulatory elements were analyzed using Plant CARE and PLACE databases. Following this, we isolated genomic DNA from the leaves of *A. thaliana* (ecotype-Columbia) by CTAB method which was used as a template to amplify the full length *PP2C*-like promoter (AT5G59220) and its various deletion constructs. Fragments were deleted from 5′ upstream of the promoter region. PCR was carried out using five different primer pairs as shown in the **Table 1**. The amplified fragments were then cloned into pGEMT easy vector. Five different promoter fragments were cloned in pBI101 (a promoter less vector) using *XbaI* and *BamHI* sites (**Figure 1**). The expression vectors containing promoter fragments of *PP2C*-like promoter were designated as full-length 1000 bp (F), 900 bp, 500 bp, 400 bp and NRM. For determining the activity of *PP2C*-like promoter, wild-type *Arabidopsis* was used as a negative control.

**Table 1:** List of primers used for amplification of PP2C-like promoter and its deletion fragments.

S.No	Sequence 5′–3′	Temperature °C
(1)	(F) Forward 5′**TGCTCTAGA**AAGTATTCACGCACCAAGGT 3′	58
(2)	900 bp 5′**TGCTCTAGA**TGTCCTTGAACACACCAAAC 3′	60
(3)	500 bp 5′**TGCTCTAGA**AAATGGTAAGGTA AATTTCCAC 3′	58
(4)	400 bp 5′**TGCTCTAGA**CACTTTAGGTCTGAGTAGTGT 3′	58
(5)	NRM 5′**TGCTCTAGA**GCGATTTAGGAGAAGTACGT 3′	58
(6)	R 5′**CGCGGATCC**TTTATATTAGCTTCTTTCACCAG 3′	60

*TCTAGA is XBa I site & inner nucleotides flanking to it*.

### *Arabidopsis* Transformation by Floral Dip Method

*Agrobacterium tumefaciens* (GV3101) containing promoter-*gusA* (β*-glucuronidase*) fusion constructs in pBI101 were used to transform *A. thaliana* plants by floral dip method (Clough and Bent, 1998). Primary transgenic explants were grown and maintained in a growth chamber at 22–23°C under 16 h light/8 h dark cycle (Murashige and Skoog, 1962). Transgenic plants obtained were then screened for integration of the promoter-*gusA* chimeric gene into the genome by PCR. Genomic DNA was isolated from leaves of kanamycin resistant *A. thaliana* plants using Qiagen DNeasy plant mini kit. PCR analysis was carried out using kanamycin specific primers and GUS specific primers.

### Hormone-Induced Treatments in Transgenic *Arabidopsis thaliana*

Transgenic *A. thaliana* plants were grown in the growth chamber under conditions specified above. Three week old plants were treated with ABA and MeJA. Leaves of transgenic *Arabidopsis* were treated with100 μM ABA, 100 μM SA, and 50 μM MeJA for 12, 24, 36, and 48 h, respectively, at room temperature for fluorometric analysis and real time PCR (qRT-PCR) for 24 and 48 h. Tissues under un-induced conditions were used as control.

### Quantitative Real-time PCR

RNA was isolated from the leaves of transgenic plants and was extracted using Qiagen RNeasy Plant Mini kit as described by the manufacturer (Qiagen, Valencia, CA, USA). cDNA strand was synthesized by priming with oligo-dT using SuperScript III reverse transcriptase (Invitrogen Carlsbad, CA, USA). PCR reactions were carried out using ABI PRISM^®^7900 HT Sequence Detection System (Applied Biosystems, Foster City, CA, USA). SYBR^®^Green was used for quantification of dsDNA. Below mentioned PCR conditions were followed for amplification of reaction volume of 10 μl: 50°C for 2 min; 95°C for 10 min; 40 cycles of 95°C for 15 s and 60°C for 1 min. SYBR^®^Green fluorescence was measured continuously during the PCR. Ubiquitin was used as internal control.

### Protein Extraction and GUS Fluorometric Analysis

Behavior of *PP2C*-like promoter and its deletion variants induced by various hormones was studied using transgenic *A. thaliana* lines. Fluorometric analysis of GUS activity was performed using 4-methylumbelliferyl-b-glucuronide (4-MUG). Extracted proteins were mixed with GUS assay buffer (2 mM 4-MUG, 50 mM sodium phosphate buffer pH 7.0, 10 mM β-mercaptoethanol, 10 mM Na_2_EDTA, 0.1% sodium lauroyl sarcosine, and 0.1% Triton X-100). Further addition of the stop buffer (0.2 M Na_2_CO_3_) stopped the progress of the reaction. 4-MUG was hydrolyzed by GUS to produce 4-methylumbelliferone fluorochrome (4-MU). GUS activity was determined using excitation wavelength of 365 nm and the emission wavelength of 455 nm. Protein concentration was determined using Bradford assay (according to the manufacturer’s instructions, Jefferson et al., 1987).

### Histochemical analysis

GUS histochemical staining of transgenic *A. thaliana* plants containing *PP2C*-like promoter -GUS fusion constructs was carried out by following the method described by Jefferson et al. (1987) for plants under ABA, SA, and JA. The results were recorded using a Canon scanner and GUS-positive plant tissues were examined with the help of a bright field microscope (Leica Q500MC, Cambridge, England).

## Results

### Analysis of *Cis* Regulatory Elements

**FIGURE 2:**
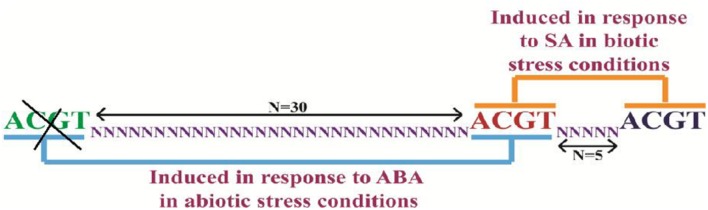
**Design of NRM construct by deletion of ACGT element, which is one of the deletion variants of *PP2C*-like promoter**. The deleted element has been highlighted in yellow color and *N* = 30 and 5 is underlined in *N*. *N* represents — nucleotides.

*In silico* analysis of *PP2C*-like promoter (accession number AT5G59220) was carried out in *A. thaliana* genome. We identified that the *PP2C*-like promoter has a distinctive genetic arrangement of ACGT elements (**Figure 2**). The *PP2C*-like promoter has three ACGT elements in close vicinity (at 665, 700, and 709, **Figure 2**). Two of them were separated by 30 nucleotides while the central ACGT element was separated by five nucleotides with another ACGT element. Mehrotra and Mehrotra (2010) have shown that two ACGT element separated by 25 nucleotides were induced in response to ABA while the one separated by five bps was induced by SA (Mehrotra et al., 2005, 2012, 2013). Multiple *cis* regulatory elements family like light regulatory elements, stress responsive elements and SA elements have been reported in PP2C-like promoter. Analysis of these putative regulatory elements by PLACE and PlantCARE databases proposed that *PP2C*-like promoter may respond to a variety of inducers such as ABA, JA, and SA signals (Prestridge, 1991; Higo et al., 1999). It can be inferred that *PP2C*-like promoter could be an inducible promoter, regulated by abiotic factors and hormones. Four homolog sequences of the pathogenesis and salt-related *cis* element GT1GMSCAM4 (GAAAAA) were found in the full-length *PP2C*-like promoter. Two homolog sequences of WBOXATNPR1 (TTGAC) were also noticed in full-length *PP2C*-like promoter. In addition, light-responsive elements such as GT1 (GRWAAW) and GATABOX (GATA) were also present. Only two homolog sequences of ABRERATCAL (MACGYGB) element, a calcium responsive *cis* element were identified in full-length *PP2C*-like promoter. *PP2C*-like promoter was characterized by making a series of 5′ deletion constructs like 900 bp, 500 bp, 400 bp and NRM, followed by analysis of *cis* regulatory using PLACE and PlantCARE databases (Higo et al., 1999; Lescot et al., 2002). In the present study, inducers like ABA, JA, and SA were used to illustrate the promoter activity in the presence and absence of these elicitors.

### Fluorometric Analysis of GUS Activity under Uninduced Condition

**FIGURE 3:**
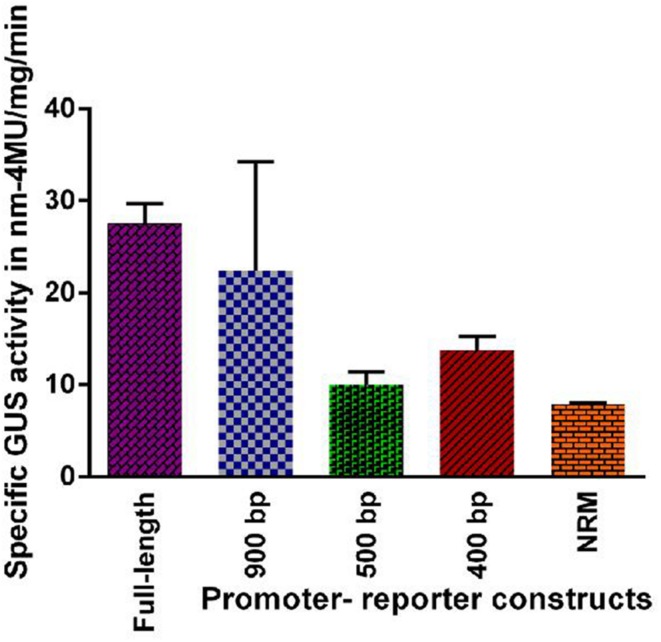
**Fluorometric assay of GUS activity in transgenic lines of *Arabidopsis* plants harboring full length and deletion constructs viz 900 bp, 500 bp, 400 bp and NRM of *PP2C*-like promoter under normal conditions.** The vertical bar indicates standard deviation.

GUS activity was estimated fluorometrically in the transgenic lines (leaves of the plant) harboring full-length and the different deletion promoter-reporter constructs of PP2C-like promoter. Results obtained are shown in **Figure 3**. As compared with the other three deletion constructs the full length promoter and 900 bp construct have higher GUS activity under non-induced conditions.

### Analysis of GUS Expression under Hormonal Treatment

**FIGURE 4:**
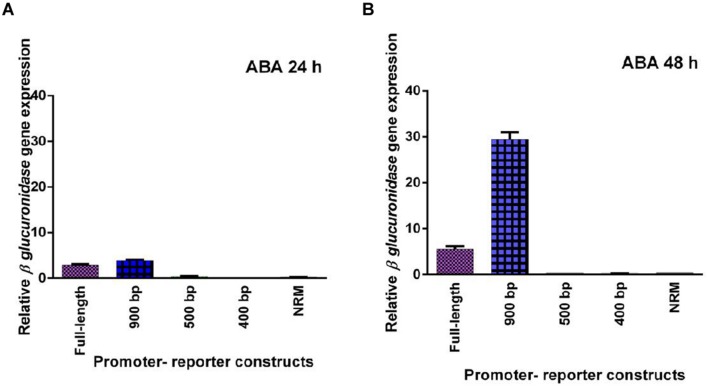
**(A,B)** Relative GUS gene expression in response to exogenous ABA - full length promoter, 900 bp, 500 bp, 400 bp, and NRM deletion constructs of *PP2C*-like promoter. The vertical bar indicates standard deviation.

**FIGURE 5:**
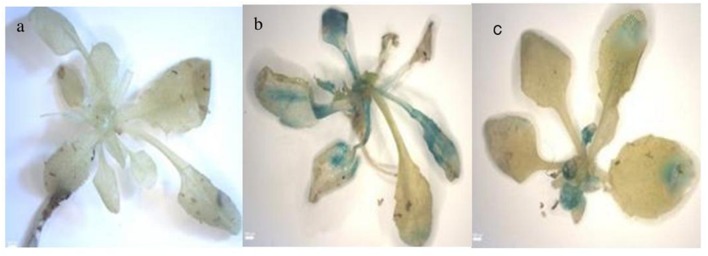
**Histochemical analysis (a) Wild type ABA treated, (b) full length ABA treated, (c) 900 bp ABA treated**.

**FIGURE 6:**
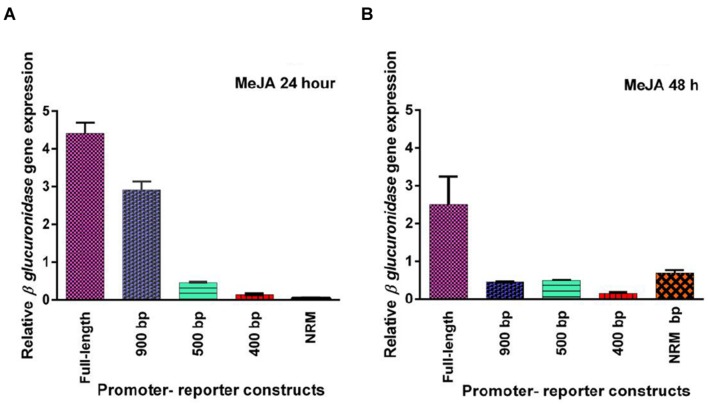
**(A,B)** Relative GUS gene expression of five *PP2C*-like promoter constructs full promoter, 900 bp deletion, 500 bp, 400 bp, and NRM deletion constructs, 24 and 48th hour after MeJA treatment. The vertical bar indicates standard deviation.

**FIGURE 7:**
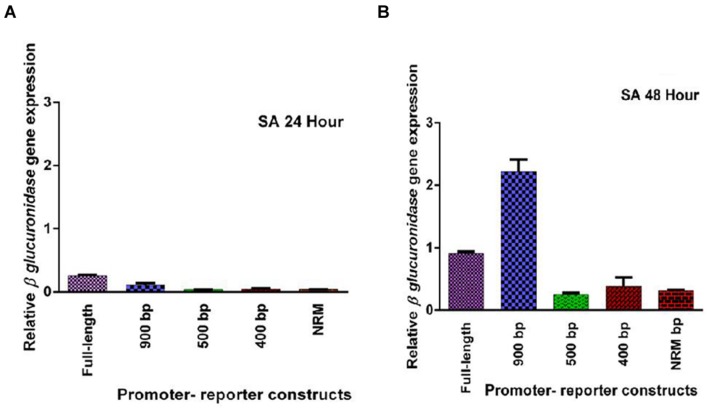
**(A,B)** Relative GUS gene expression of five *PP2C*-like promoter constructs including full promoter, 900 bp deletion, 500 bp, 400 bp, and NRM deletion constructs, at 24 and 48th hour after SA treatment. The vertical bar indicates standard deviation.

Five promoter- reporter cassettes of *PP2C*-like promoter viz full length, 900 bp, 500 bp, 400 bp and NRM were differently expressed upon exogenous application of ABA (100 μM). RNA was extracted from leaves after 24 and 48 h ABA (dissolved in autoclaved milli Q water) treatment. Gene expression was quantified using real-time PCR, and the relative amounts of transcripts were then calculated and normalized with *ubiquitin* mRNA. GUS activity of ABA (100 μM) treated plants was fluorometrically analyzed for the period of 12, 24, 36, and 48 h. In leaves, full-length and 900 bp deletion constructs of *PP2C*-like promoter responded significantly to ABA, whereas other deletion constructs of *PP2C*-like promoter showed relatively little GUS gene expression (**Figures 4A,B**). The 900 bp deletion construct has shown enhanced β-*glucuronidase* gene expression than the full length promoter after 48 h of treatment. Rest other three deletion construct responded weakly, i.e., almost no expression was observed even after 24 and 48 h of ABA treatment. GUS protein activity was assayed and the results are shown in **Figure 4** and histochemical data as shown in **Figure 5**. Change in the GUS activity of the other three promoter- reporter cassettes, i.e., 500 bp, 400 bp and NRM; was observed only at 12th hour and consequently there was no change. 900 bp construct responded positively to ABA amongst all deletion promoter cassettes unlike the three deletion cassettes which showed negligible GUS activity. Under MeJA (dissolved in ethanol) treatment the β-*glucuronidase* gene expression level was found to be high in the full length promoter and 900 bp deletion constructs in leaves after 24 h (**Figures 6A,B**). After 48th hour of treatment, β-*glucuronidase* gene expression decreased in the full length and 900 bp deletion constructs (**Figures 6A,B**). Other cassettes had lower β-*glucuronidase* gene expression at 24 and 48th hour treatment. After 24 h of SA (dissolved in autoclaved milliQ water) treatment, less gene expression was observed in leaves in case of all the promoter constructs. The 900 bp promoter constructs induced at 48th hour gave high GUS expression in leaves, while in case of other constructs a slight increase in gene expression was observed (**Figures 7A,B**). In comparison to non-induced condition, no change in the GUS activity was observed (**Figure 3**). This indicated that *PP2C*-like promoter was not inducible in response to SA.

## Discussion

According to PLACE and Plant CARE databases, ABA responsive element (ABRE), drought responsive elements (DRE) or MYC and MYB recognition sites are the pivotal *cis*-regulatory elements present in the *PP2C*-like promoter sequence (Higo et al., 1999; Lescot et al., 2002). Further *cis* elements also exist as dyads within a specific distance from one another, for example, ABRE–ABRE or ABRE-CE required for ABA response (Gomez-Porras et al., 2007). It has been observed that the deletion of -1.9 kb region of the promoter of *gbss*1 gene (granule-bound starch synthase 1 from wheat) led to the reduction in GUS activity suggesting the presence of vital enhancers and *cis* acting elements (Kluth et al., 2002). In one of the studies on β-*phaseolin* gene promoter, it has been observed that some A/T rich sequences possibly act as general enhancers of expression in the developing embryo (Bustos et al., 1989) and light-regulated photosynthesis gene because of its positive role in gene expression (Lam et al., 1990). For initiation of transcription, CAAT (enhancer *cis* element) and TATA sites were used for synthesizing inducible promoters (Roychoudhury and Sengupta, 2009). The elements like ACGTERD1, DRE1COREZMRAB17 (ACCGAGA), and ABRELATERD1 are found to be present in *PP2C*-like promoter (**Figure 2B**). Six copies of ACGTATERD1 elements are present in full length and 900 bp of *PP2C*-like promoter, four in 500 and 400 bp constructs whereas only three copies are present in NRM construct.

In the present study, GUS expression in the 900 bp construct was much more than the one in full length promoter suggesting the involvement of certain negative regulatory sequences in the full length promoter. Also, 900 bp construct showed a substantial increase after 48 h of ABA treatment which is supposedly due to its late responsive nature. Decrease in the copy number of CAAT elements or other important *cis* elements could be one of the possible reasons for the reduced gene expression observed in the three deletions constructs. From the earlier studies, activity of OsAct2 (actin 2 promoter from rice) could be defined by the presence of a negative regulator (+96 and +274 within the intron) which represses *OsAct2* expression. Conversely, positive regulators i.e., the two *CAAT-boxes* found to be present (between -448 and -445, and positions -635 and -632) enhances *OsAct2* expression (de los et al., 2015). From this scrutiny it can be inferred that the full length and 900 bp deletion construct of *PP2C*-like promoter is an ABA inducible promoter. This also demonstrates that the deleted elements are required for optimal gene expression. In NRM, ACGT_N30_ ACGT was deleted (**Figure 2B**) which resulted in the loss of expression which could be due to the deletion of the ACGT copies and their CEs. This indicates that possibly appropriate ABRE complex was not forming in the smaller deletion constructs like -500 bp, -400 bp, and NRM since there was no response to ABA treatment in these constructs. A study by Hong and Hwang (2009) found -593 bp deletion construct to be insensitive to ABA treatment, although this construct had ABA responsive bZIP and MYB binding sites. The results suggest that *PP2C*-like promoter deletion of -100 bp, i.e., 900 bp promoter-reporter cassettes was sufficient to drive GUS gene expression. Decrease in the MeJA induced gene expression might be due to the removal of some transcription factor binding sites (**Figures 6A,B**). Variation in activity may be due to the decrease in enhancer *cis*-acting elements viz CAAT elements (A/T rich region). In another study, it was observed that, -1210 to -886 bp was sufficient for MeJA-induced GUS activity in *OsPMCa^2^*^+^*ATPase* promoter, whereas the -519 bp deletion from *OsPMCa^2^*^+^*ATPase* promoter showed a reduced MeJA-responsive promoter activity in leaves (Kamrul-Huda et al., 2013). The GCC-box (JA -responsive element) was not observed in the *PP2C*-like promoter region, which could also be one of the reasons for weak induction shown by the *PP2C*-like promoter constructs in response to MeJA. Decrease in activity was observed when two ACGT elements separated by N5 were placed 100 nucleotides away from *Pmec* (Mehrotra et al., 2005). This was observed in all the cassettes of *PP2C*-like promoter. Inspite of the presence of a TTGAC element within the *OsPMCa^2^*^+^*ATPase* promoter region located at -1261 bp there was no induction in response to SA (Kamrul-Huda et al., 2013). In this study, the promoter constructs containing these *cis* elements were activated by SA treatment. In the deletion constructs, W-box and TGACG copy number decreases, resulting in the reduced activity.

*Cis* regulatory elements are involved in a variety of regulatory networks, affecting each other’s role and their respective position in different promoters (Wray et al., 2003). Protein–protein interactions also play an important role in the gene expression. For instance, SCOF-1 protein (soybean cold inducible factor-1) interacts with SGBF-1 (soybean G-box binding bZIP transcription factor) in response to cold stress (Kim et al., 2001).

Varied intensity of GUS gene expression was noticed in different transgenic lines (Moon and Callahan, 2004). GUS mRNA levels were found to be very low in the plants transformed with the shortest promoter constructs of *ACO-GUS* whereas a increase was detected in the transgenic line bearing -610 promoter- reporter construct. Transgenic plants with the three (-2919 to -2141, -1319 to -901, and the first 403 bp) longer promoter-reporter constructs have shown a significant increase in the amount of mRNA (Moon and Callahan, 2004).

Some of the possible reasons for this difference could be attributed to different transgene integration and variation in transgene copy number. *Cis* elements essential for activation in response to ABA, JA, and SA, are detected mostly in full length and 900 bp constructs. It can be proposed that due to multiple levels of gene regulation (viz transcriptional, post-transcriptional, translational, and post-translational level) in some subsets, there is a weak correlation between mRNA and protein levels (Walling et al., 1986). The data obtained in this study could be used to design inducible promoters which might be of great help to Agricultural Biotechnology.

## Author Contributions

RM conceptualized the study and provided resources for the present study. PB conducted all the major experiments and partially wrote the manuscript. CS wrote the manuscript partially and was involved in multiple discussions with a few suggestions. AA helped in conduction of induction experiments at NABI, Mohali and SM gave critical input and was involved in multiple discussion and reviewed the manuscript.

## Conflict of Interest Statement

The authors declare that the research was conducted in the absence of any commercial or financial relationships that could be construed as a potential conflict of interest.

## Abbreviations

AB: abscisic acid
bp: base pair
JA: jasmonic acid
*PP2C*: protein phosphatase 2C
Me ja: methyl jasmonate
SA: salicylic acid
